# An Investigation of Vitamin Levels Status in the Serum of Children in China

**DOI:** 10.1155/2018/6592757

**Published:** 2018-12-11

**Authors:** Yu-juan Chen, Miao Liu, Cui-yin Mao, Shu-hua Zhang

**Affiliations:** ^1^School of Life Science and Technology, Changchun University of Science and Technology, Changchun, China; ^2^Hospital Affiliated to Changchun University of Chinese Medicine, Changchun, China; ^3^Department of Cardiology, China-Japan Union Hospital, Changchun, China

## Abstract

This study was to investigate the vitamin levels in the serum of children in the Northeast of China and some factors associated with the vitamin levels. The sera were obtained from 2096 normal children aged 1 month to 12 years in the Northeast of China. Vitamins A and E were detected using their sera by HPLC, and the level of vitamin D was detected by LC-MS. The 20 overweight children were chosen from the 7-8 age range and took additional vitamins studies for extra 3 months. The data were analyzed by IBM SPSS Statistics 21. The average levels of vitamins A, D, and E in sera samples from the 2096 children were 0.2715mg/L (95%CI, 0.2715-0.2791mg/L), 26.2848*μ*g/L (95%CI, 25.7900-26.7786*μ*g/L), and 8.6137mg/L (95% CI, 8.5077-8.7198mg/L), respectively. The percentages of vitamins A, D, and E deficiency were 19.61%, 47.47%, and 0.62%, respectively. For 20 children from the VDD or VAD groups, after giving them more VA and VD for 3 months, the levels of VA and VD in the sera were increased significantly; however, the average BMI has barely changed. According to our finding, vitamin D deficiency was severe, vitamin A deficiency was common, and vitamin E was insufficient in the sera of children in the Northeast of China. The levels of vitamins A, D, and E were correlated with age and overweight.

## 1. Introduction

A vitamin is an organic compound and a vital nutrient that an organism requires in limited amounts. Vitamins have diverse biochemical functions. Some have hormone-like functions as regulators of cell and tissue growth and differentiation, such as some forms of vitamin A; and some are regulators of mineral metabolism, such as vitamin D (VD); others function as antioxidants, e.g., vitamin E (VE).

Vitamin A (VA) is a vital micronutrient involved in several biochemical activities crucial for regular biological purpose, including vision and immunocompetence. The sufficient concentration of VA is VA≥0.3mg/L, VA<0.2mg/L is VA deficiency (VAD), and 0.2mg/L≤VA<0.3mg/L was defined as VA insufficiency (VAI) [1]. Vitamin A deficiency (VAD) was associated with increased death and severity of illness from respiratory and gastrointestinal disease. VAD is a common form of micronutrient deficiency, globally affecting 33.3% of preschool-age children, and 0.9% with night blindness [2]. Abrha* et al*. reported that night blindness and Bitot's spots were major nutritional problems in Asgede-Tsimbla rural district, north Ethiopia [3], though neonatal vitamin A supplementation had no effect on serum vitamin A levels at six weeks of age [4].

The worldwide prevalence of vitamin D deficiency (VDD) and vitamin D insufficiency (VDI) in children increased. Soesanti* et al*. reported that there is high prevalence of VDI in healthy children aged 7-12 years in Indonesia. Girls have increased risk compared to boys [5]. Fowlie* et al*. found that VDI may play a role in peanut allergy [6]. VDD effectively predicts increased risk for childhood asthma [7]. The sufficient concentration of VD is VD≥75nmol/L (36.8*μ*g/L), VD<50nmol/L (24.5*μ*g/L) is VD deficiency (VDD), and 50nmol/L≤VD<75nmol/L was defined as VD insufficiency (VDI) [8–10]. A recent study showed that VDD and VDI were prevalent among infants, preschool children, school children, and adolescents in Southeastern China [11].

Vitamin E (VE) refers to a group of compounds that include both tocopherols and tocotrienols. *α*-Tocopherol, the most biologically active form of vitamin E, is one of the most common forms of vitamin E in the diet. Vitamin E has many biological functions, the antioxidant function being the best known [12]. Other functions include enzymatic activities, gene expression, and neurological function(s) [13, 14]. Vitamin E deficiency (VED) is not prevalent, and vitamin E insufficiency (VEI) is not common. The sufficient concentration of VE is VE≥7mg/L, VE<4mg/L is VE deficiency (VED), and 4mg/L≤VE<7mg/L was defined as VE insufficiency (VEI) [1]. Li* et al*. reported that the serum VE average level was 10.09±2.76 mg/L, and rate of insufficiency was 13.4% in Beijing for 67 healthy children [1].

Vitamins A, D, and E play a significant role in life as an important nutrition, but there is seldom investigation of vitamin levels in children in China, especially in the Northeast of China. The aim of this study was to investigate the vitamin levels and the relationship of some factors, such as overweight, age, sex, and living environment.

## 2. Materials and Methods

### 2.1. Subjects and Samples Collection

2096 children in the Northeast of China were examined with regard to their nutrition and health in 2017, in order to investigate vitamins A, D, and E and their health in this study. More than 3000 children were in the investigated plan, and 2096 children's parents consented to participation in this study. Because some analysis for this investigation was not free, some children did not agree to contribute 2mL blood. Children aged from 1 month to 12 years were chosen as the study population, including breast feeding children, preschool children, and school children. The regional ethic committee approved the study. Participation was voluntary and informed consent was obtained from all parents of participating children. Fasting blood samples were obtained by venipuncture from the antecubital vein in the morning for the next day after questioning. Blood samples were collected from 2096 children in hospital from the cities as follows: Changchun, Baicheng, Jiaohe, Jilin, Songyuan, and Siping. All of the six cities are in the Northeast of China.

### 2.2. Serum Extract and Analysis by LC-MS

As previously reported [15], fasting blood samples were centrifuged at 1000 rpm, for 10 min after collecting, and then the sera were stored at -20°C till analysis. Before analyzing, the serum was kept at room temperature for 10min, and 100 *μ*L of serum was mixed with 100 *μ*L of acetonitrile with internal standard and then centrifuged at 10000 rpm for 10 min. The internal standards of vitamins A, D_2_, D_3_, and E were vitamin A acetate, vitamin E acetate, 25-hydroxyvitamin D_2_-d_3_, and 25-hydroxyvitamin D_3_-d_6_, separately. Then the supernatants were collected and extracted by 200 *μ*L hexane. Then 100 uL hexane extract was dried by stream of nitrogen gas, and the residue materials were dissolved in 50 *μ*L methane and then analyzed by High Performance Liquid Chromatography (HPLC) or Liquid Chromatography-Mass Spectra (LC-MS). The method was validated as previously, in terms of lower limit of quantification, accuracy, precision, linearity, and extraction recovery [15].

### 2.3. Data Collection

All the 2096 children's parents completed the health questionnaire which assessed different parameters, including sex, age, residential area, body height, and weight. BMI was calculated as body mass in kilograms divided by height in meters squared (kg/m^2^). Overweight was defined as BMI ≥85th percentile and normal weight as BMI between 5th and 84th percentile [16].

### 2.4. Data Analysis

All results were analyzed using IBM SPSS Statistics 21 for statistical analysis. Frequency distribution of VA, VD, and VE concentrations was described using column graph with normal distribution curve. For the levels of VA, VD, and VE, continuous variables were presented as mean ± SD while categorical variables were presented as percentage rate. Pearson correlation analysis was used to describe the correlations of VA, VD, VE, and age. Statistical significance was set at P< 0.01.

## 3. Results

### 3.1. Method Validation

The signal and noise heights are used to calculate signal-to-noise ratio, and the results of lower limit of quantification of vitamins are shown in Table 1. The calibration curve was prepared by plotting the peak area ratios of vitamin to internal standard versus the concentrations ratios of vitamin to internal standard. The calibration curve shows good linearity over the range 10-1000 *μ*g/L, 1-80 *μ*g/L, 10-200 ug/L, and 0.5-80 mg/L for VA, VD_2_, VD_3_, and VE, respectively. And the results of linearity are shown in Table 1. The extraction recovery was calculated by comparing the peak intensity of vitamin internal standard extracted from serum samples with these compounds directly injected to HPLC or LC-MS. All the averages of extraction recoveries of VA, VD_2_, VD_3_, and VE were determined during 90%-105% (as shown in Table 1).

### 3.2. VA, VD, and VE Status of Children

**Figure 1:**
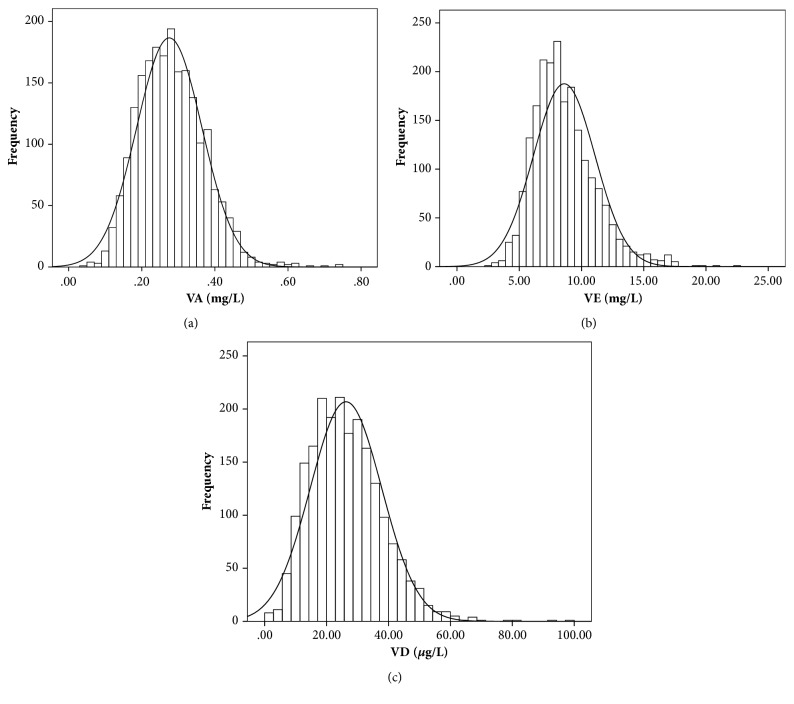
Frequency distribution of VA, VD, and VE concentrations in children's sera samples. In the children's sera samples, the mean VA level was 0.2753mg/L (95% CI, 0.2715-0.2791 mg/L); the mean VD level was 26.2848 *μ*g/L (95% CI, 25.7900-26.7786); the mean VE level was 8.6137 mg/L (95% CI, 8.5077-8.7198mg/L), N=2096.

In this survey, total number of the children aged from 1 month to 12 years was 2096, in Northeast of China, 2017. There were 963 girls and 1132 boys. The number of children aged from 1 month to 3 years, 4 to 6 years, and 7 to 12 years was 1129, 543, and 424, respectively. The specific number of children in this survey is shown in Table 2. The frequency distribution of VA, VD, and VE concentrations is shown in Figure 1, and all the levels of vitamins are in the normal distribution. The mean levels of VA, VD, and VE were 0.2753mg/L (95% CI, 0.2715-0.2791 mg/L), 26.2848 *μ*g/L (95% CI, 25.7900-26.7786), and 8.6137 mg/L (95% CI, 8.5077-8.7198mg/L), respectively.

The number of VAD was 411, and that of VAI was 861. The ratio of VAD and VAI was 19.61% and 41.08%, respectively. As shown in Table 2, in the three age groups, the ratio of VAI was the highest in 0-3 age group, and the ratio of VAI in the 7-12 age groups was the lowest. The number of VDD was 995, and that of VDI was 742. The ratios of VDD and VDI were 47.47% and 35.04%, respectively. Only 17.49% of children investigated were VD sufficient. Among the 2096 children, only 13 were VED. And the number of VEI was 562, and the ratio was 26.81%. There was no significant difference of mean levels of VA, VD, and VE between boys and girls. However, the girls' VDD was 8.77% higher than the boys. And the boys' VEI was 6.21% higher than the girls. In the three age groups, the ratio of VEI (37.38%) among the aged 4-6 group was the highest; the ratio of VAI (43.31%) and VDI (40.21%) among the aged 0-3 group was the highest, and that of VDD (33.66%) and VEI (20.55%) was the lowest; the ratio of VAI (37.74%), VAD (16.04%), and VDI (25.94%) among the aged 7-12 group was the lowest, and that of VDD was the highest.

### 3.3. Factors Associated with Vitamin Levels

**Figure 2:**
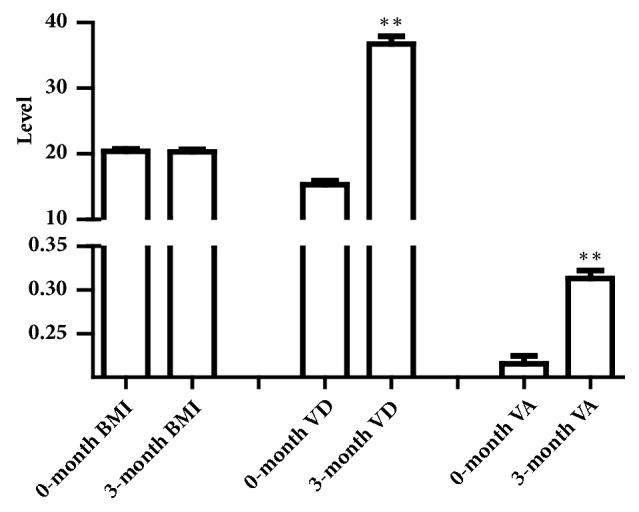
Vitamin levels and BMI data in the serum of trail children during 3 months (n=20, 7-8 years old).

**Figure 3:**
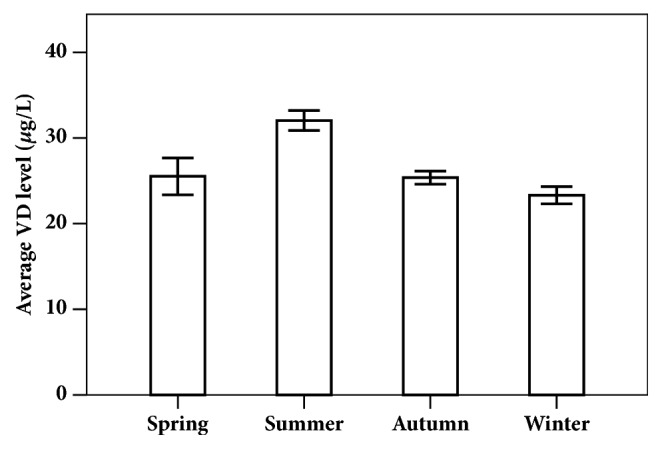
The average VD level in children's sera samples in different seasons.

In this survey, the factors associated with vitamin levels among children in Northeast of China are showed in Table 3. The vitamin levels of children in urban area were higher than those in rural area. Among the 2096 children, there were 355 children who were overweight identified by BMI. The percentage of overweight children in the VAD group (32.36%) was 2.6 times that of VA sufficiency group (12.26%). And the percentage of overweight children in the VDD group (20.20%) was also higher than that of VD sufficiency group (16.43%). The results are shown in Table 3. When giving the overweight children enough VA and VD for 3 months, the average of VA and VD level in the serum was sufficient after the trail, but the BMI was no different as shown in Figure 2. As shown in Table 4, Pearson correlation was positive and significant at the 0.01 level (2-tailed) between VA and VE, VD, and age. The positive correlation of VE and VD was significant at the 0.01 level (2-tailed), too. The negative correlation of VE and age was significant at the 0.01 level (2-tailed). The negative correlation of VD and age was significant at the 0.01 level (2-tailed), too. In this survey, the seasonal variation in VD level was significant, shown in Figure 3, especial in summer and winter.

## 4. Discussion

In China, most of parents do not want to have blood tests for their small baby (less than one month), unless the baby is sick. So the investigated children aged from 1 month to 12 years. The cases in this study were normal children; most of them did not take supplemental vitamins. They took supplemental vitamins when they were identified as being vitamin insufficient and their doctors suggested them to take them. Some of them took calcium formula which contained VD, and the recommended dose of VD was 100 IU/d.

There was no significant difference on vitamin levels status (VA, VD, and VE) between girls and boys in this study, VA levels increased with age increasing. The results were consistent with previous reports [17]. VE and VD levels decreased with age increasing. There were some reasons: some pregnant women did not take enough VA, 17.05% of pregnant women had VAD [15], and VA was insufficient in their breast milk [1, 18]; small babies did not intake enough VA from their mothers' breast milk. It was also possible because small children grew quicker and consumed more VA, and they did not get enough vitamins from food, and the serum VA levels were lower during the first few years. With the babies growing up, they could intake more and more kinds of food and obtained more VA from foods. All these caused the percentage of VAI to be the highest in the 0-3 age group, among all groups. In this study, VDD in females was higher than males. The reasons are boys more likely play outdoors, and girls tend to play in rooms, and the frequency of sunlight exposure is significantly higher in boys than girls [19]. Due to abusing ripening agent and hormones nowadays, scientists think there are fewer nutrients in foods than before, especially vitamins and minerals. Children need to intake more vitamins besides those from foods.

Through the investigation of the vitamin levels in children and interview, the results showed the relationship between vitamin levels and some factors, such as age, sex, overweight, and living environment. The significant factor was overweight, so the 3 months' trail was designed. 20 overweight children who were VAI /VAD or VDI/VDD were advised to take enough VA and VD formula every day for 3 months. After 3 months, the VA and VD levels in the serum increased obviously, though the average of BMI had barely changed. These results suggested that taking enough vitamins was not useful in reducing the body weight in short time. Vitamins A and D are fat-soluble and stored in adipose tissue. One predominant hypothesis in the human literature is that vitamin D is sequestered in the greater body fat mass of obese individuals as compared with lean cohorts, and this leads to reduced bioavailability and circulating serum concentrations of 25(OH)D [20]. So the overweight children need to take more VA and VD from diet; if the diet does not have enough fat-soluble vitamins, they need to take enough additional vitamin formula to maintain the vitamin level in the serum for bioavailability. More researches were needed to study the relationship between overweight and the level of vitamins. Nowadays, living environment does not play a significant role in the vitamin levels in children, because the gap between urban and rural area is not as big as decades ago in the Northeast of China. As a previous study in pregnant women, the correlation of VE and VD was significant at the 0.01 level (2-tailed). VA and VE, VA, and VD were the same. As the results indicated, vitamins levels in the children serum may interact with each other.

To determine if there was seasonal variation in VD level, the data of children's sera sample were analyzed by time. In the Northeast of China, spring is from March to May, summer is from June to August, autumn is from September to November, and winter is from December to February of the following year. As shown in Figure 3, the level in the children's sera samples was higher in summer than in winter; the possible reason was that the children were exposed to more sunlight when they played outside in summer; and they played indoors in winter, because it was too cold outside.

## 5. Conclusions

According to our findings, vitamin D deficiency was severe, vitamin A deficiency was common, and vitamin E was insufficient in children serum in the Northeast of China; the levels of vitamins A, D, and E in the sera of children in Northeast of China were correlated with age. Sufficiency of vitamins may have relationship with body weight, but taking enough VA and VD was not useful for overweight people to reduce body weight in a short time period.

## Figures and Tables

**Table 1:** The results of method validation of VA and VE determined by HPLC and VD determined by LC-MS.

	**LLQ (ng/mL)**	**RSD**	**linearity**	**extraction recovery/RSD**
VA	40	2.74%	y=0.3037x+0.002079 (R2=0.9997)	92-103%/4.67%
VD2	0.5	3.45%	y=0.361x+0.000459 (R2=0.9958)	90-105%/5.71%
VD3	0.5	5.78%	y=0.465x+0.011 (R2=0.9943)	90-105%/6.24%
VE	30	2.89%	y=0.2923x+0.1388 (R2=0.9991)	91-104%/4.01%

**Table 2:** VA, VD, and VE levels of Children (1 month to 12 years) in Northeast of China, 2017.

	**Total**	**Child sex**		**Age group (year)**		
	**(n=2096)**	**Female(n=963)**	**Male(n=1132)**	**0-3(n=1129)**	**4-6(n=543)**	**7-12(n=424)**
VA (mg/L)	0.275±0.090	0.276±0.089	0.275±0.089	0.268±0.088	0.280±0.093	0.288±0.087
VD (*μ*g/L)	26.285±11.559	25.442±11.381	27.023±11.634	29.818±12.307	22.655±9.007	21.524±8.947
VE (mg/L)	8.614±2.422	8.904±2.605	8.369±2.334	9.191±2.747	7.914±2.00	7.973±1.791
VAD (n)	411 (19.61%)	200(20.77 %)	211(18.64%)	232(20.55%)	111(20.44%)	68(16.04%)
VAI (n)	861 (41.08%)	378(39.25%)	482(42.58%)	489(43.31%)	212(39.04%)	160(37.74%)
VDD (n)	995 (47.47%)	503(52.23%)	492(43.46%)	380(33.66%)	315(58.01%)	290(68.40%)
VDI (n)	742 (35.40%)	330(34.27%)	412(36.40%)	454(40.21%)	178(32.78%)	110(25.94%)
VED (n)	13 (0.62%)	4(0.42%)	9(0.80%)	9(0.80%)	2(0.47%)	2(0.47%)
VEI (n)	562 (26.81%)	226(23.47%)	336(29.68%)	232(20.55%)	203(37.38%)	127(29.95%)

**Table 3:** Factors associated with vitamin levels among children in Northeast of China, 2017 (n=2096, 1 month to 12 years).

**n**		**factors**			
		**Overweight (n=355)**		**Urban**	**Rural**
		**Yes**	**No**	**n=1083 (**%**)**	**n=1013 (**%**)**
VA	Sufficiency (n=824)	101 (12.26%)	723 (87.74%)	546 (50.42%)	278 (27.44%)
	VAI (n=861)	121 (14.05%)	740 (85.95%)	379 (35.00%)	482 (47.58%)
	VAD (n=411)	133 (32.36%)	278 (67.64%)	158 (14.59%)	253 (24.98%)
VD	Sufficiency (n=359)	48(13.37%)	311(86.63%)	175 (16.16%)	184 (18.16%)
	VDI (n=742)	106(14.29%)	636(85.71%)	417 (38.50%)	325 (32.08%)
	VDD (n=995)	201(20.20%)	794(79.80%)	491 (45.34%)	504 (49.75%)
VE	Sufficiency (n=1521)	210 (13.81%)	1311 (86.19%)	840 (77.56%)	681 (67.23%)
	VEI (n=562)	141 (25.09%)	421 (74.91%)	238 (21.98%)	324 (31.98%)
	VED (n=13)	4 (30.77%)	9 (69.23%)	5 (0.46%)	8 (0.79%)

**Table 4:** Correlations of VA, VD, and VE levels and age.

		**VA**	**VE**	**VD**	**Age**
VA	Pearson correlation	1	.322*∗∗*	.105*∗∗*	.103*∗∗*
	Sig.(2-tailed)		.000	.000	.000
	N	2096	2096	2096	2096
VE	Pearson correlation	.322*∗∗*	1	.232*∗∗*	-.273*∗∗*
	Sig.(2-tailed)	.000		.000	.000
	N	2096	2096	2096	2096
VD	Pearson correlation	.105*∗∗*	.232*∗∗*	1	-.311*∗∗*
	Sig.(2-tailed)	.000	.000		.000
	N	2096	2096	2096	2096
Age	Pearson correlation	.103*∗∗*	-.273*∗∗*	-.311*∗∗*	1
	Sig.(2-tailed)	.000	.000	.000	
	N	2096	2096	2096	2096

*∗∗*Correlation is significant at the 0.01 level (2-tailed).

## Data Availability

The data used in this article are restricted. The data used to support the findings of this study are available from the corresponding author on reasonable request. The data are restricted, because most of the parents of the children did not want to disclose information about their children.

## Abbreviations

BMI: Body mass index
HPLC: High Performance Liquid Chromatography
LC-MS: Liquid Chromatography-Mass Spectra
VA: Vitamin A
VAD: Vitamin A deficiency
VAI: Vitamin A insufficiency
VD: Vitamin D
VDD: Vitamin D deficiency
VDI: Vitamin D insufficiency
VE: Vitamin E
VED: Vitamin E deficiency
VEI: Vitamin E insufficiency.
